# Pru du 1, the Bet *v* 1‐homologue from almond, is a major allergen in patients with birch pollen associated almond allergy

**DOI:** 10.1002/clt2.12177

**Published:** 2022-08-08

**Authors:** Stefan Kabasser, Nadja Crvenjak, Stefanie Schmalz, Tanja Kalic, Christine Hafner, Pawel Dubiela, Aleksandra Kucharczyk, Stanislawa Bazan‐Socha, Mateusz Lukaszyk, Heimo Breiteneder, Christian Radauer, Merima Bublin

**Affiliations:** ^1^ Institute of Pathophysiology and Allergy Research Center of Pathophysiology Infectiology and Immunology, Medical University of Vienna Vienna Austria; ^2^ Department of Dermatology University Hospital St. Pölten Karl Landsteiner University of Health Sciences St. Pölten Austria; ^3^ Karl Landsteiner Institute for Dermatological Research St. Pölten Austria; ^4^ Department of Regenerative Medicine and Immune Regulation Medical University of Bialystok Bialystok Poland; ^5^ Department of Internal Diseases, Pneumonology, Allergology Clinical Immunology, Military Institute of Medicine Warsaw Poland; ^6^ Department of Internal Medicine Jagiellonian University Medical College Krakow Poland; ^7^ Department of Lung Diseases and Tuberculosis Medical University of Bialystok Bialystok Poland

**Keywords:** almond allergy, Bet *v* 1‐homologue, oral allergy syndrome, pollen food syndrome, tree nut allergy

## Abstract

**Background:**

Almond allergy is common and can manifest in two different forms. Primary almond allergy has been reported to be associated with sensitization to almond legumin Pru du 6. In birchendemic regions, there is a link between birch‐pollinosis which is likely based on a cross‐reactive Bet v 1 homologue, a yet unidentified allergen in almond. Therefore, we sought to identify and characterize a Bet v 1‐homologue in almond.

**Methods:**

The expression of a Bet v 1 homologue in almond kernels was confirmed by mass spectrometry. The recombinant protein was produced in *Escherichia coli* and its cross‐reactivity and allergenic potency was analyzed by IgE quantitative and competitive ELISA, immunoblotting and basophil histamine release using sera from 17 almond allergic patients.

**Results:**

The identified Bet v 1 homologue received the designation Pru du 1.0101. Pru du 1.0101 bound IgE from 82 % of almond allergic patients. Bet v 1 was able to inhibit IgE‐binding to rPru du 1 by 100%, while rPru du 1 inhibited IgE binding to rBet v 1 by 48%. Pru du 1.0101 activated basophils, though 100‐ to 1000‐fold higher concentrations were required for maximum activation in comparison to rBet v 1.

**Conclusion:**

Considering the strong inhibition capacity and higher allergenic potency of Bet v 1, the results provide compelling evidence for primary sensitization to Bet v 1 in case of birch pollen associated almond allergy. Combining Pru du 6 and Pru du 1 in diagnostic approaches may help to discriminate between primary and birch‐pollen associated almond allergy.

## To the Editor,

Almond allergy is common and can manifest in two different forms. Primary almond allergy, which presents with moderate to severe symptoms, has been reported to be associated with sensitization to the legumin Pru du 6.^1^ In birch‐endemic regions, there is a link between birch‐pollinosis and mild secondary almond allergy which is likely, based on a cross‐reactive Bet v 1 homologue, a yet unidentified allergen in almond.^2^, ^3^ Accurate diagnosis of almond allergy is complicated by the poor correlation between sensitization and clinical reactivity. An improvement of molecular almond allergy diagnosis could help in the interpretation of in vitro IgE testing and guide the selection of patients for oral food challenges. Therefore, the aim of this study was to characterize and assess the IgE reactivity of a Bet v 1‐homologue in almond, which represents a missing component in the diagnosis.

**FIGURE 1:**
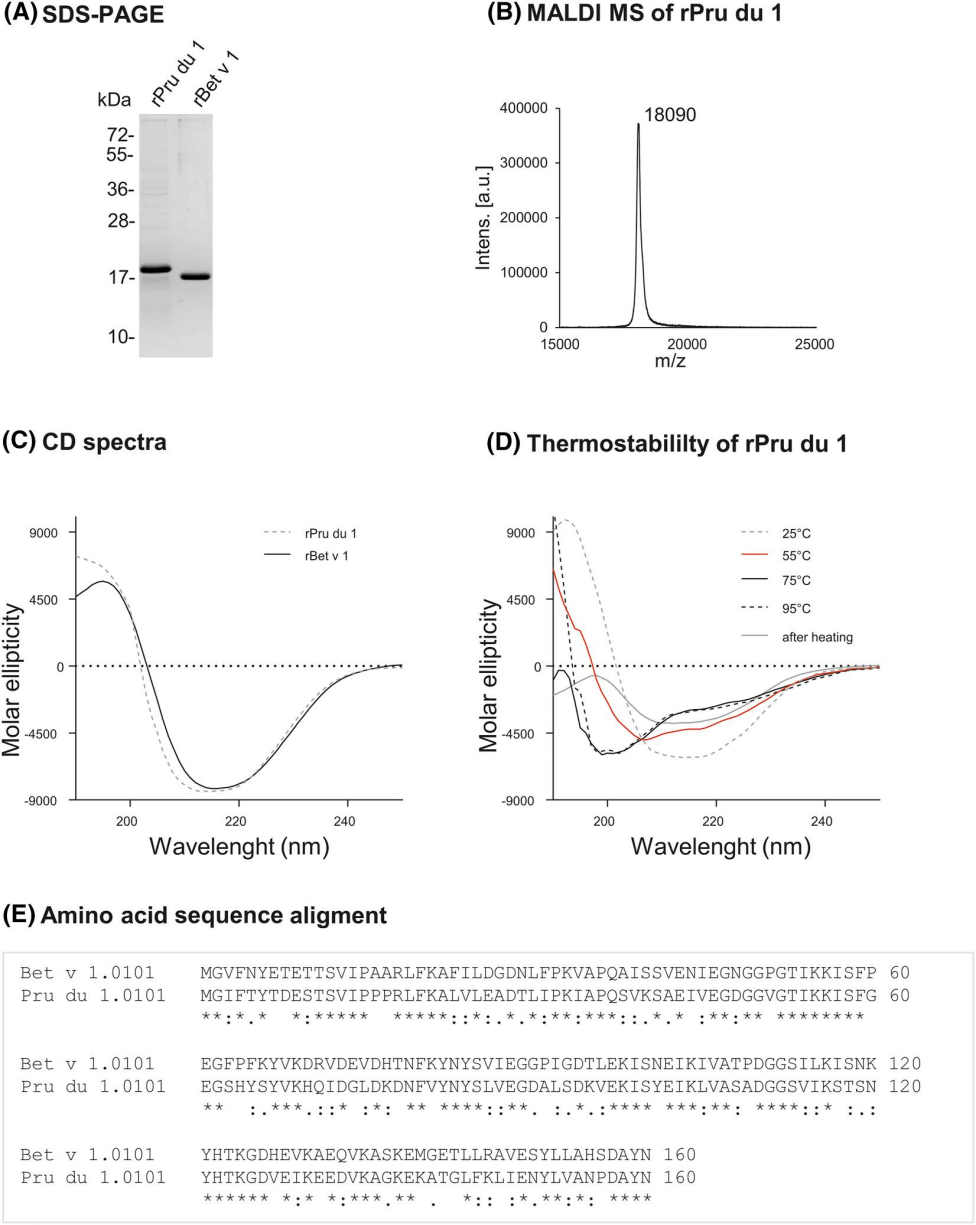
Physicochemical characterization of rPru du 1. (A) Coomassie blue‐stained 15% sodium dodecylsulfate dolyacrylamide gel electrophoresis (SDS‐PAGE) of rPru du 1 and rBet v 1 under reducing conditions. (B) Matrix‐assisted laser desorption/ionization time‐of flight mass spectrometric (MALDI MS) analysis of rPru du 1. (C) Circular dichroism (CD) spectra of rBet v 1 and rPru du 1 measured at room temperature. (D) Thermal denaturation of rPru du 1 upon heating to 95°C. (E) Comparison of amino acid sequences of Bet v 1 and Pru du 1. Stars (*) indicate full residue conservation, “:” indicate strongly similar, and “.” indicate weakly similar amino acid properties

By tandem mass spectrometry of proteins extracted from almond kernels, we identified peptides matching to the sequence of one of seven previously identified *Pru du 1* genes, namely Pru du 1.06 A.^4^ For further characterization, we produced the protein recombinantly in *E. coli* (Figure 1A). The molecular mass of rPru du 1 (18,090.82 Da, Figure 1B) was in accordance with the calculated mass of 18,087.26 Da. The circular dichroism spectrum revealed that the secondary structure of rPru du 1 was nearly identical to that of rBet v 1, showing a folded protein with mixed *α*‐helical and *ß*‐strand structures (Figure 1C). In a thermostability assay, Pru du 1 started losing structural integrity at 55°C and could not refold after heating to 95°C (Figure 1D). The allergen, now included in the World Health Organization/International Union of Immunological Societies Allergen Nomenclature Database by the name Pru du 1.0101, shows 56% amino acid sequence identity to Bet v 1 (Figure 1E). In immunoblot, rPru du 1 was recognized by two anti‐Bet v 1‐specific antibodies (BIP‐1^5^ and BV16^6^), indicating shared epitopes in the highly conserved glycine‐rich region (Figure S1).

**FIGURE 2:**
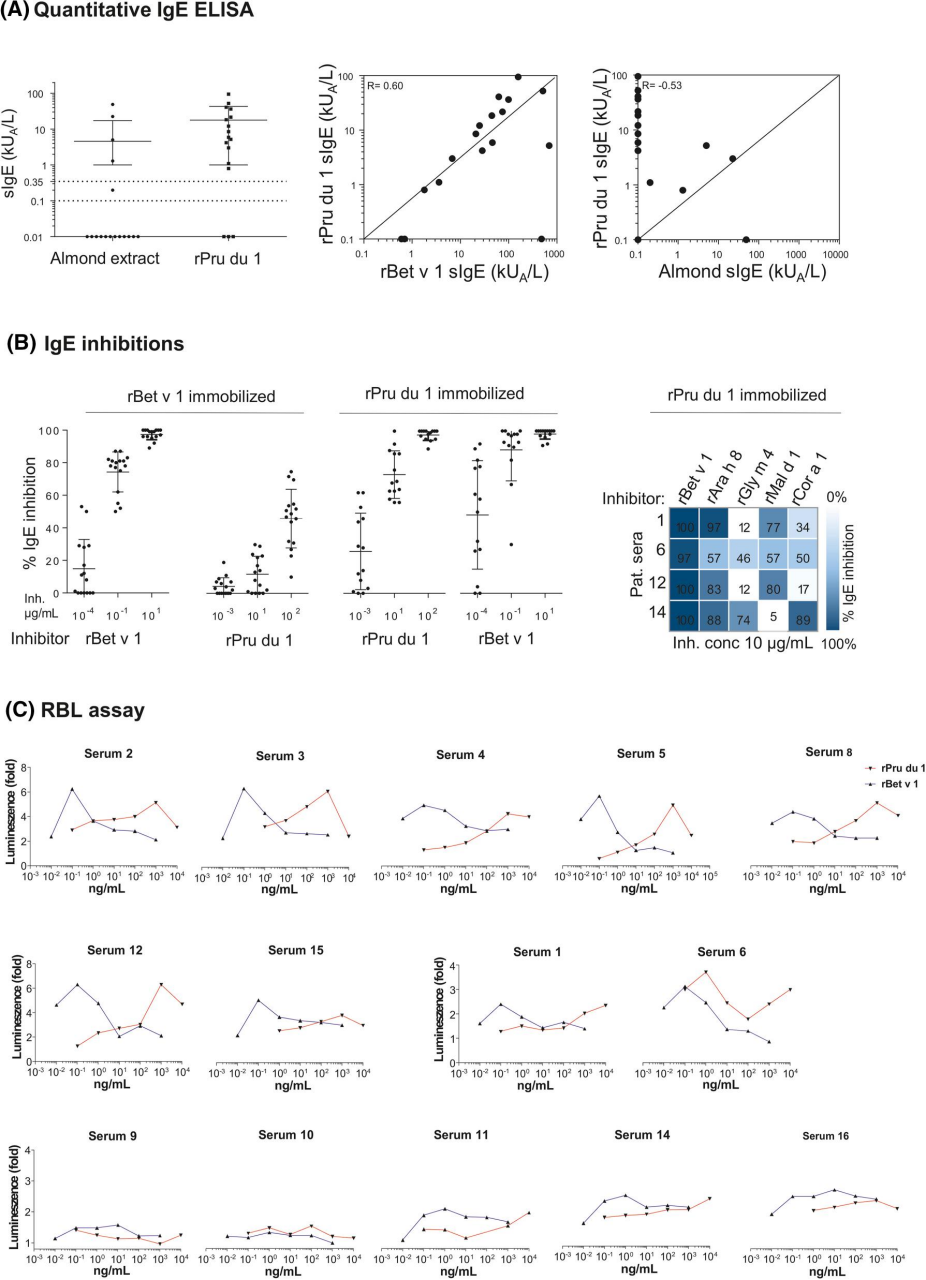
Immunological characterization of rPru du 1. (A) Specific IgE to almond extract and rPru du 1 of sera from 17 almond allergic patients and correlation between sIgE levels to rPru du 1, almond extract and rBet v 1 in almond allergic patients (Spearman correlation). (B) IgE inhibition ELISA. Sera were inhibited with increasing concentrations of rBet v 1 (0.0001–10 μg/ml) and rPru du 1 (0.001–100 μg/ml). Horizontal bars indicate medians ± interquartile ranges. Inhibitions with other allergens were performed at 10 μg/ml. *Inh. conc.: Inhibitor concentration. (C) Basophil activation assay with RS‐ATL8 rat basophil leukemia (RBL) cells sensitized with sera from 14 patients (Table S1)

Among 17 almond allergic patients with mainly oral allergy syndrome, 29% had sIgE to almond extract, whereas 82% were positive to rPru du 1 (Table S1 and Figure 2A). IgE reactivity to rPru du 1 showed no correlation with reactivity to almond extract (Spearman coefficient: −0.53) (Figure 2A). It seems that the Bet v 1 homologue is underrepresented in the extract, similar to what has been previously observed for hazelnut and walnut.^7^, ^8^ In comparison, a correlation was observed with IgE reactivity to rBet v 1 (Spearman coefficient: 0.60). In IgE immunoblot, pre‐incubation of a serum pool with rBet v 1 completely abolished IgE binding to rPru du 1, however rPru du one only partially inhibited IgE reactivity to rBet v 1 (Figure S1). Similarly, in an IgE ELISA, rBet v 1 was able to inhibit IgE reactivity to rPru du 1 by 100%, while rPru du 1 inhibited IgE binding to rBet v 1 by a median of 48% (Figure 2B). Recombinant Ara h 8 was able to inhibit IgE reactivity to rPru du 1 by 86%, followed by rMal d 1 (74%), rCor a 1 (42%), and rGly m 4 (29%). In a rat basophil leukemia cell activation assay with humanized rat basophilic leukemia cells, rPru du 1 was able to activate the cells sensitized with almond allergic patients' sera, though higher concentrations (up to 1 μg/ml) were required for maximum activation in comparison to rBet v 1 (Figure 2C).

Summarizing, we for the first time biochemically and immunologically characterized a Bet v 1‐homologous allergen in almond kernels. Combining Pru du 6 and Pru du 1 in diagnostic approaches may help to discriminate between primary and birch‐pollen associated almond allergy.

## AUTHOR CONTRIBUTION

**Stefan Kabasser**: Investigation; Lead, Methodology; Lead, Writing – original draft; Equal, Writing – review & editing; Equal, **Nadja Crvenjak**: Investigation; Supporting, Methodology; Supporting, **Stefanie Schmalz**: Methodology; Supporting, **Tanja Kalic**: Investigation; Supporting, Methodology; Supporting, **Christine Hafner**: Investigation; Supporting, Resources; Equal, Supervision; Supporting, Writing – review & editing; Supporting, **Pawel Dubiela**: Formal analysis; Supporting, Resources; Supporting, **Aleksandra Kucharczyk**: Resources; Supporting, **Stanislawa Bazan‐Socha**: Resources; Supporting, **Mateusz Lukaszyk**: Resources; Supporting, **Heimo Breiteneder**: Resources; Supporting, **Christian Radauer**: Formal analysis; Supporting, Methodology; Supporting, Supervision; Supporting, **Merima Bublin**: Conceptualization; Lead, Funding acquisition; Lead, Investigation; Equal, Methodology; Equal, Resources; Lead, Supervision; Lead, Writing – original draft; Equal, Writing – review & editing; Equal

## CONFLICT OF INTEREST

The authors have no conflicts of interest to declare.

## Supporting information

Supplementary Material

## Data Availability

The data that supports the findings of this study are available in the supplementary material of this article.
