# Supplementary material for: Pru du 1, the Bet v 1‐homologue from almond, is a major allergen in patients with birch pollen associated almond allergy
Source: Clin Transl Allergy. 2022 Aug 8;12(8):e12177. doi: 10.1002/clt2.12177 (PMC9358727; doi:10.1002/clt2.12177)
Supplement: Supplementary file 1 — Supplementary Material [file CLT2-12-e12177-s001.docx]

**Supplementary material**

**Patient’s sera**

For our study, 17 sera from patients with almond allergy were used (Table S1). Almond allergy was diagnosed based on a convincing history and a positive skin prick test to commercial almond extract (Diater Laboratorios Madrid, Spain) or positive almond ImmunoCAP (Thermo Fisher Scientific, Uppsala, Sweden) with sIgE ≥ 0.35 kU/L. The patients included in the study with a diagnosed tree pollen allergy and/or grass pollen allergy suffered from seasonal allergic rhinoconjunctivitis. The use of clinical data and serum samples for this study was approved by the ethics committees of the Medical University of Vienna, Austria and the Medical University in Poznan, Poland. Signed informed consent was obtained from all patients.

**Preparation of almond protein extract**

Almond kernels (species *Prunus dulcis*) were ground in a household blender and proteins were extracted at 4 °C for 1 h by stirring with 5 volumes of PBS containing protease inhibitor cocktail tablets (Roche Molecular Biochemicals). The homogenate was centrifuged (21 000 x g, at 4 °C for 1 h) and the supernatant was used for subsequent experiments.

**Identification of a Pru du 1.06A in almond kernels by Nano-LC ESI Orbitrap MS/MS**

Protein identification was performed at the VetCore Facility for Research (Veterinary University of Vienna, Vienna, Austria), as described previously ^1^.

**Heterologous expression of rPru du 1.0101 and other Bet v 1-homologues in *E.coli* and protein purification**

The DNA sequence of Pru du 1.06A (UniProt accession number: B6CQS9), in the following named Pru du 1, was used for recombinant protein expression. Codon optimization, gene synthesis and insertion of the sequence into the pET28b(+) expression vector (Novagen

Madison, WI) were performed by BioCat. The protein was fused to a 6xHis tag and expressed in the *E.coli* strain NiCo21[DE3] (Catalog #C2529, New England Biolabs). Expression was performed using autoinduction *E. coli* culture medium at 30 °C, as previously described ^2^. After overnight expression, the cells were pelleted and disrupted by high-pressure microfluidics in 50 mM sodium phosphate buffer, pH 7.5, containing protease inhibitor cocktail tablets (Roche Molecular Biochemicals). Purification of recombinant Pru du 1 was performed by nickel chelate affinity chromatography and *E. coli*-derived chitin binding contaminants were subsequently removed using chitin affinity chromatography according to the manufacturer's instructions (Catalog #C2529, New England Biolabs). The identity was checked by matrix-assisted laser desorption/ionization-time of flight (MALDI-TOF) mass spectrometry (Bruker Daltonics, Bremen, Germany). Recombinant Bet v 1.0101, Ara h 8.0101, Gly m 4.0101, Mal d 1.0201, and Cor a 1.0401 were expressed and purified as described previously ^3-5^.

**CD spectroscopy and thermostability assay**

CD spectra of recombinant Pru du 1 (0.25 μg/μL in 10 mM sodium phosphate buffer, pH 7.5) were measured in the range of 190 to 260 nm on a Jasco J-810 spectropolarimeter (Jasco International Co.) at room temperature using a 1 mm path length quartz cell. The thermal stability of Pru du 1 was assessed by stepwise heating to 95°C (2 °C/min) and subsequent cooling to 25°C. Spectra represent the average of three accumulations collected at 50 nm/min with a 2 s time constant, 0.5 nm resolution, and sensitivity of ±100 mdeg.

**IgE ELISA and dose-dependent inhibition ELISA**

IgE ELISA and quantitation of sIgE were performed as previously described^1^. The sIgE concentration (kU_A_/L) was calculated using a factor of 1U=2.4 ng/mL. In order to study IgE inhibition in a dose-dependent manner, sera were pre-incubated for 2 h with rBet v 1 (0.0001-10 µg/mL) or rPru du 1 (0.001-100 µg/mL). Other Bet v 1-homologues were used at a concentration of 10 µg/mL.

**Immunoblotting**

For immunoblotting, membranes were incubated after blocking with pooled sera of almond allergic patients. Alternatively, the monoclonal anti-Bet v 1-antibodies BV16 (Absolute Antibody Ltd, Redcar, UK) and BIP1^6^ were used. For detection of bound antibodies by enhanced chemiluminescence (ECL), membrane strips were incubated with a 1:5000 diluted HRP conjugated anti-human IgE antibody (KPL, Gaithersburg, MD, USA) or a 1:20000 diluted HRP conjugated anti-mouse IgG antibody (KPL, Gaithersburg, MD, USA). As negative controls, stripes were incubated with buffer and the two secondary antibodies. For IgE inhibition immunoblotting, the serum pool was pre-incubated for 2 h at room temperature with rPru du 1 or rBet v 1 at 50 µg/mL before adding to the membranes.

**RBL assay**

The RBL assay was carried out as described by Nakamura et al ^7^. Briefly, cells were sensitized by adding patients’ sera (diluted 1:10 – 1:20) to the supernatant. After overnight incubation, the cells were stimulated (3 h at 37 °C) by the addition of rPru du 1 (0.1-10 000 ng/mL) or rBet v 1.0101 (0.01-1000 ng/mL). Luciferase expression levels were determined by incubation of cell lysates with luciferin. Luminescence values were converted to stimulation indexes (SI). SI = sample value/spontaneous release value.

**TABLE S1.** Clinical characteristics and sIgE levels to almond extract and allergens (rPru du 1 and rBet v 1) of 17 almond allergic patients used for the study.

| **Patient no.** | **Age (y)** | **Sex** | **Symptoms to**  **almond** | **SPT or ImmunoCAP sIgE (kU/L) to almond** | **Other food allergies** | **Inhalative allergies** | **qELISA sIgE (kU/L)** | | | | |  |
| --- | --- | --- | --- | --- | --- | --- | --- | --- | --- | --- | --- | --- |
|  |  |  |  |  |  |  | **Almond** | **Pru du 1** | **Bet v 1** | **Pru du 6** | **Pru p 3*** | |
| 1 | 43 | F | OAS | 0.71 kU/L | hazelnut | alder, birch, hazel | 0.0 | 18.4 | 44.9 | 0.0 | 0.0 |  |
| 2 | 20 | F | OAS, URT*, AE | + | eggs, milk | birch, hazel | 0.0 | 94.9 | 157.7 | 0.0 | 0.0 |  |
| 3 | 51 | F | OAS | + | apple, carrot, hazelnut | alder, birch, mugworth | 0.0 | 21.7 | 74.5 | 0.0 | 0.0 |  |
| 4 | 51 | M | OAS | + | apple, carrots, cherries, hazelnut, nectarine, walnut | alder, birch | 0.0 | 4.2 | 28.4 | 0.0 | 0.0 |  |
| 5 | 38 | M | OAS | + | apple, carrot, cherries, hazelnut, nectarine, peach, pear, walnut | alder, birch, grass, oak | 0.0 | 52.2 | 513.6 | 0.0 | 0.0 |  |
| 6 | 15 | F | OAS | 0.9 kU/L | fruits, peanut, soy | birch | 0.0 | 36.3 | 99.2 | 0.0 | 0.0 |  |
| 7 | 10 | F | URT | 0.36 kU/L | cashew, hazelnut, walnut | NA | 0.0 | 5.9 | 46.0 | 0.0 | 0.0 |  |
| 8 | 58 | M | URT*, AE | + | apple, orange | birch, hazel | 0.0 | 12.1 | 24.9 | 0.0 | 0.0 |  |
| 9 | 33 | M | OAS, AE | + | NA | birch | 0.0 | 0.0 | 0.7 | 0.0 | 0.0 |  |
| 10 | 41 | F | OAS, URT* | + | seafood | none | 0.0 | 0.0 | 0.6 | 0.0 | 0.0 |  |
| 11 | 49 | F | OAS | + | apple, celery, cherries, potato | alder, birch, hazel | 1.3 | 0.8 | 1.8 | 0.0 | 0.0 |  |
| 12 | 49 | F | OAS | 0.56 kU/L | hazelnut, peanut | birch | 0.0 | 40.8 | 62.5 | 0.0 | 0.0 |  |
| 13 | 25 | M | OAS | ++ | cashew, hazelnut, peanut, pine nut, walnut | none | 49.0 | 0.0 | 480.4 | 8.0 | 9.6 |  |
| 14 | 34 | F | URT* | 0.45 kU/L | apple, apricot, celery, hazelnut, peanut, soy | birch | 0.0 | 8.5 | 21.0 | 0.0 | 0.0 |  |
| 15 | 21 | F | URT*, AE | + | cashew, macadamia, seeds | birch, grass | 5.0 | 5.2 | 692.9 | 3.6 | 0.55 |  |
| 16 | 26 | M | AE | +++ | hazelnut, pea, peanut, walnut | birch | 22.6 | 3.0 | 6.8 | 0.0 | 0.0 |  |
| 17 | 75 | M | URT*, AE, | + | apple, hazelnut, peanut, strawberry, walnut | birch | 0.2 | 1.1 | 3.6 | 0.0 | 0.0 |  |

AE: angioedema; OAS: oral allergy syndrome; NA: not available; SPT. Skin prick test; URT*: urticarial localized to the circumoral zone; *Pru p 3 was measured as representatives of an almond non specific Lipid Transfer Protein (nsLTP).

**rBet v 1**

**rPru du 1**

**rPru du 1**

**B)**

**A)**


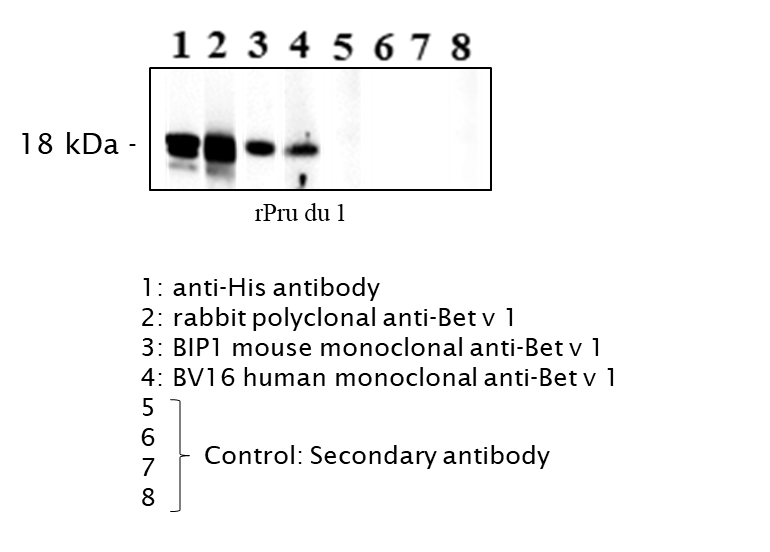

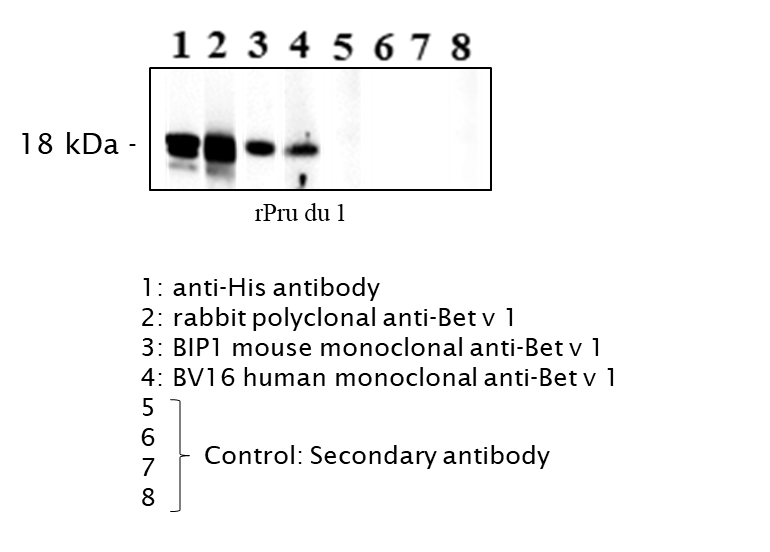

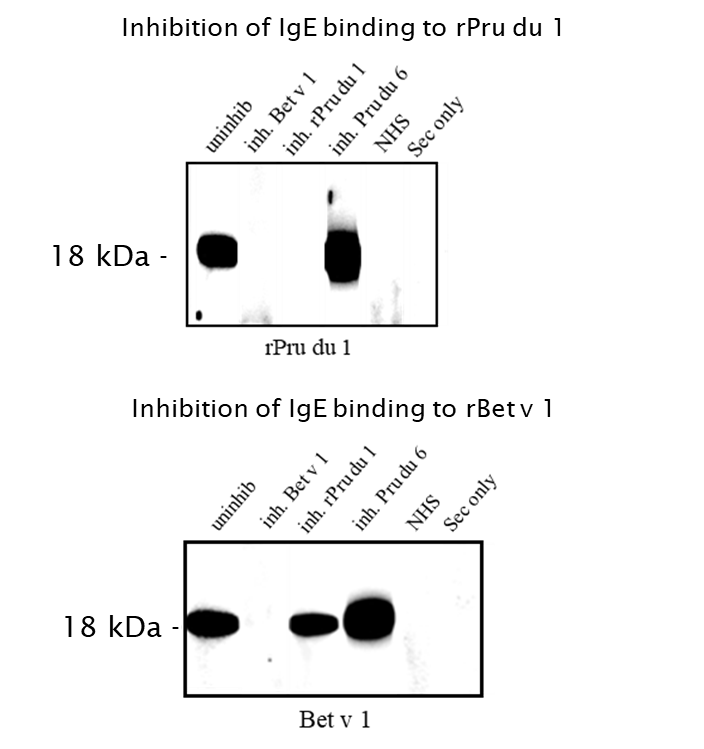

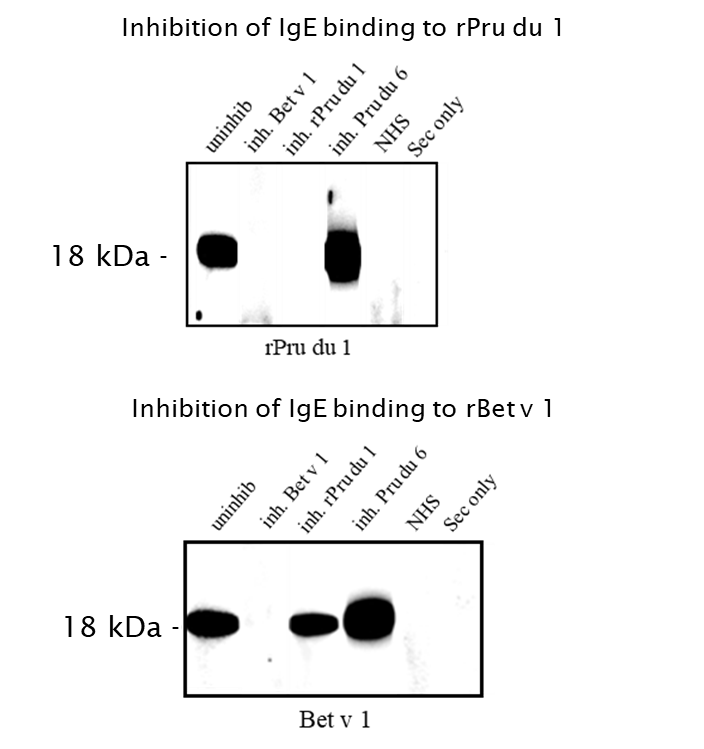


uninhib.

inh. rBet v 1

inh. rPru du 1

uninhib.

inh. rPru du 1

inh. rBet v 1

BIP-1

BV16

neg. control

neg. control

kDa

18-

18-

kDa

**Figure S1. IgE immunoblot.** A) Detection of rPru du 1 by Bet v 1-specific monoclonal antibodies BIP-1 and BV16. B) Pooled sera (No. 2, 3, 5) were tested for IgE reactivity to rPru du 1 and rBet v 1. Uninhib: Uninhibited serum pool. For inhibitions, the serum pool was inhibited with rPru du 1 or rBet v 1 at 50 µg/mL.

**References**

1. Kabasser S, Hafner C, Chinthrajah S, et al. Identification of Pru du 6 as a potential marker allergen for almond allergy. *Allergy.* 2021;76(5):1463-1472.

2. Studier FW. Protein production by auto-induction in high density shaking cultures. *Protein Expr Purif.* 2005;41(1):207-234.

3. Gepp B, Lengger N, Bublin M, Hemmer W, Breiteneder H, Radauer C. Chimeras of Bet v 1 and Api g 1 reveal heterogeneous IgE responses in patients with birch pollen allergy. *J Allergy Clin Immunol.* 2014;134(1):188-194.

4. Hurlburt BK, Offermann LR, McBride JK, Majorek KA, Maleki SJ, Chruszcz M. Structure and function of the peanut panallergen Ara h 8. *J Biol Chem.* 2013;288(52):36890-36901.

5. Oberhuber C, Ma Y, Marsh J, et al. Purification and characterisation of relevant natural and recombinant apple allergens. *Mol* ***N****utr* ***F****ood* ***R****es.* 2008;52(2):S208-219.

6. Jarolim E, Tejkl M, Rohac M, et al. Monoclonal antibodies against birch pollen allergens: characterization by immunoblotting and use for single-step affinity purification of the major allergen Bet v I. *Int Arch Allergy Appl Immunol.* 1989;90(1):54-60.

7. Nakamura R, Uchida Y, Higuchi M, Tsuge I, Urisu A, Teshima R. A convenient and sensitive allergy test: IgE crosslinking-induced luciferase expression in cultured mast cells. *Allergy.* 2010;65(10):1266-1273.
